# Supplementary material for: Design, Synthesis, and Biological Evaluation of HDAC Degraders with CRBN E3 Ligase Ligands
Source: Molecules. 2021 Nov 29;26(23):7241. doi: 10.3390/molecules26237241 (PMC8658794; doi:10.3390/molecules26237241)

Design, Synthesis, and Biological Evaluation of HDAC Degrad-ers with CRBN E3 Ligase Ligands

Yingxin Lu [1,†], Danwen Sun [2,†], Donghuai Xiao [1], Yingying Shao [2], Mingbo Su [2], Yubo Zhou [2], Jia Li [2,*], Shulei Zhu [1,*] and Wei Lu [1,*]

[1] Shanghai Engineering Research Center of Molecular Therapeutics and New Drug Development, School of Chemistry and Molecular Engineering, East China Normal University, 3663 North Zhongshan Road, Shanghai 200062, China; luyingxin_roy@163.com (Y.L.); cpu_zsl@163.com (D.X.); wlu@chem.ecnu.edu.cn (W.L.); slzhu@chem.ecnu.edu.cn (S.Z.)

[2] National Center for Drug Screening, State Key Laboratory of Drug Research, Shanghai Institute of Materia Medica, Chinese Academy of Sciences, 189 Guo Shoujing Road, Shanghai 201203, China; dan-wensun1@163.com (D.S.); yingying_shao1226@163.com (Y.S.); mbsu@simm.ac.cn (M.S.); ybzhou@simm.ac.cn (Y.Z.)

* Correspondence: jli@simm.ac.cn (J.L.); slzhu@chem.ecnu.edu.cn (S.Z.); wlu@chem.ecnu.edu.cn (W. L.);

† These authors contribute equally.

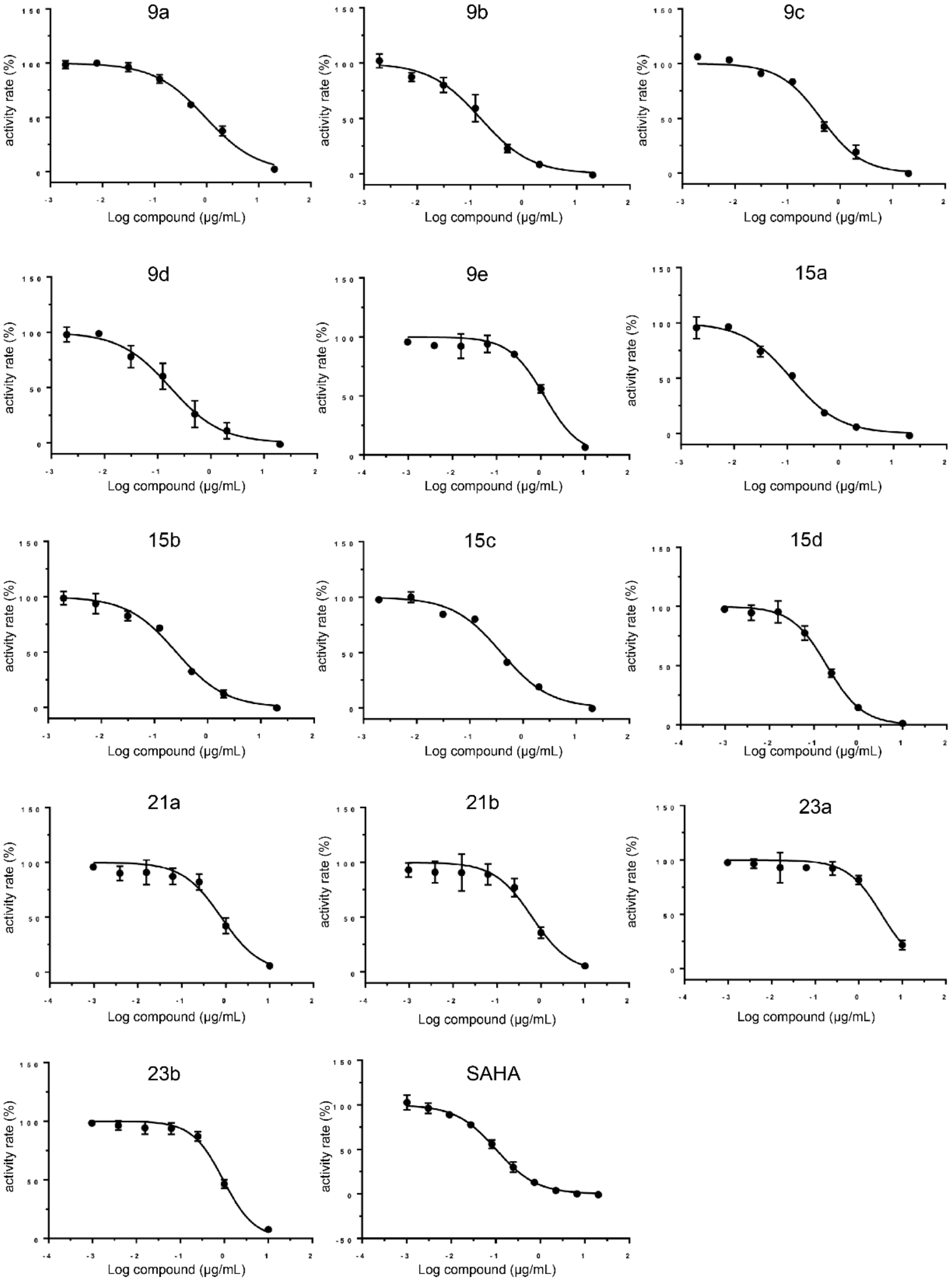

Figure S1. IC50 curve of synthesized PROTACs and SAHA against HDAC1.

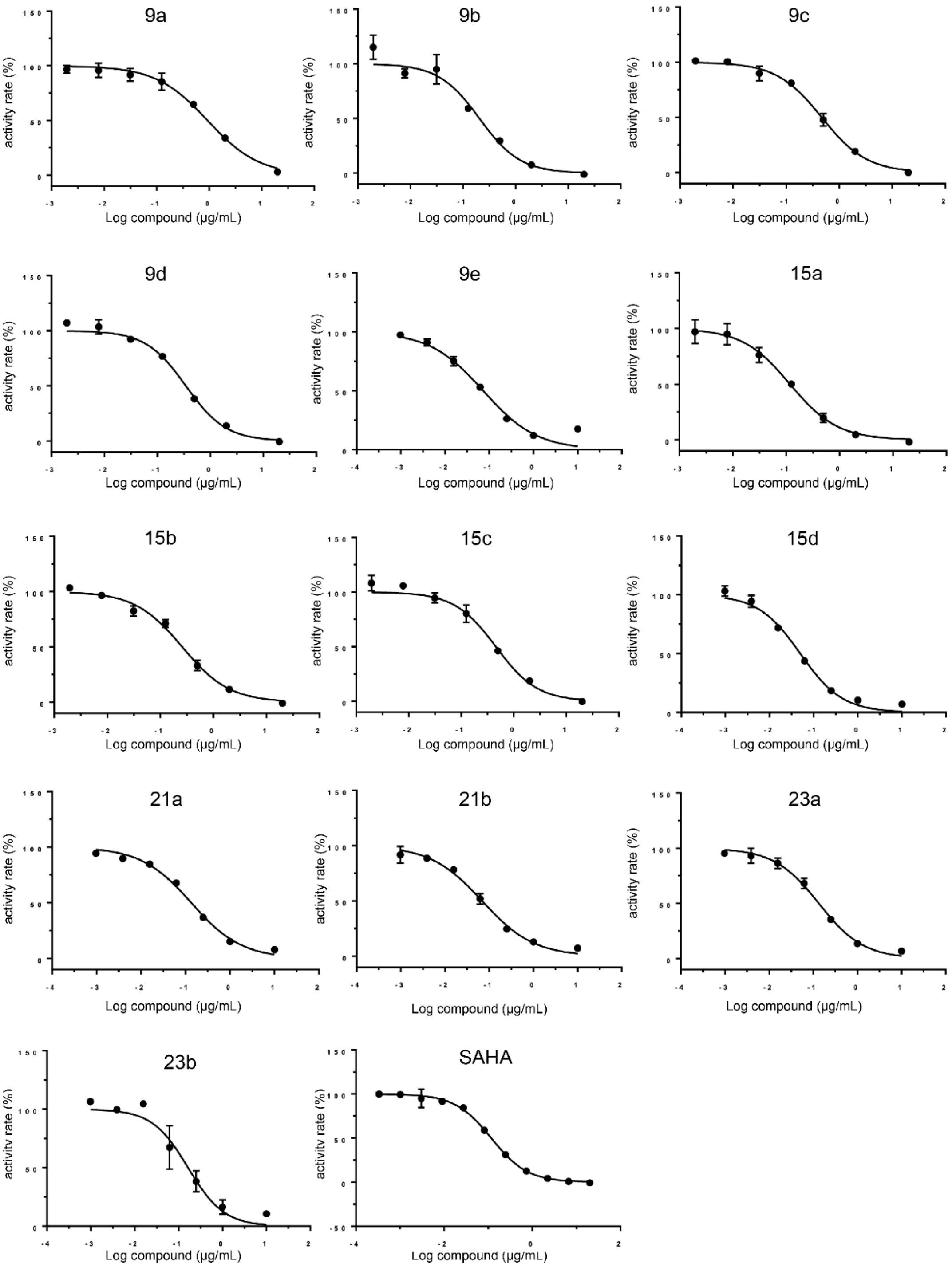

Figure S2. IC_{50} curve of synthesized PROTACs and SAHA against HDAC3.

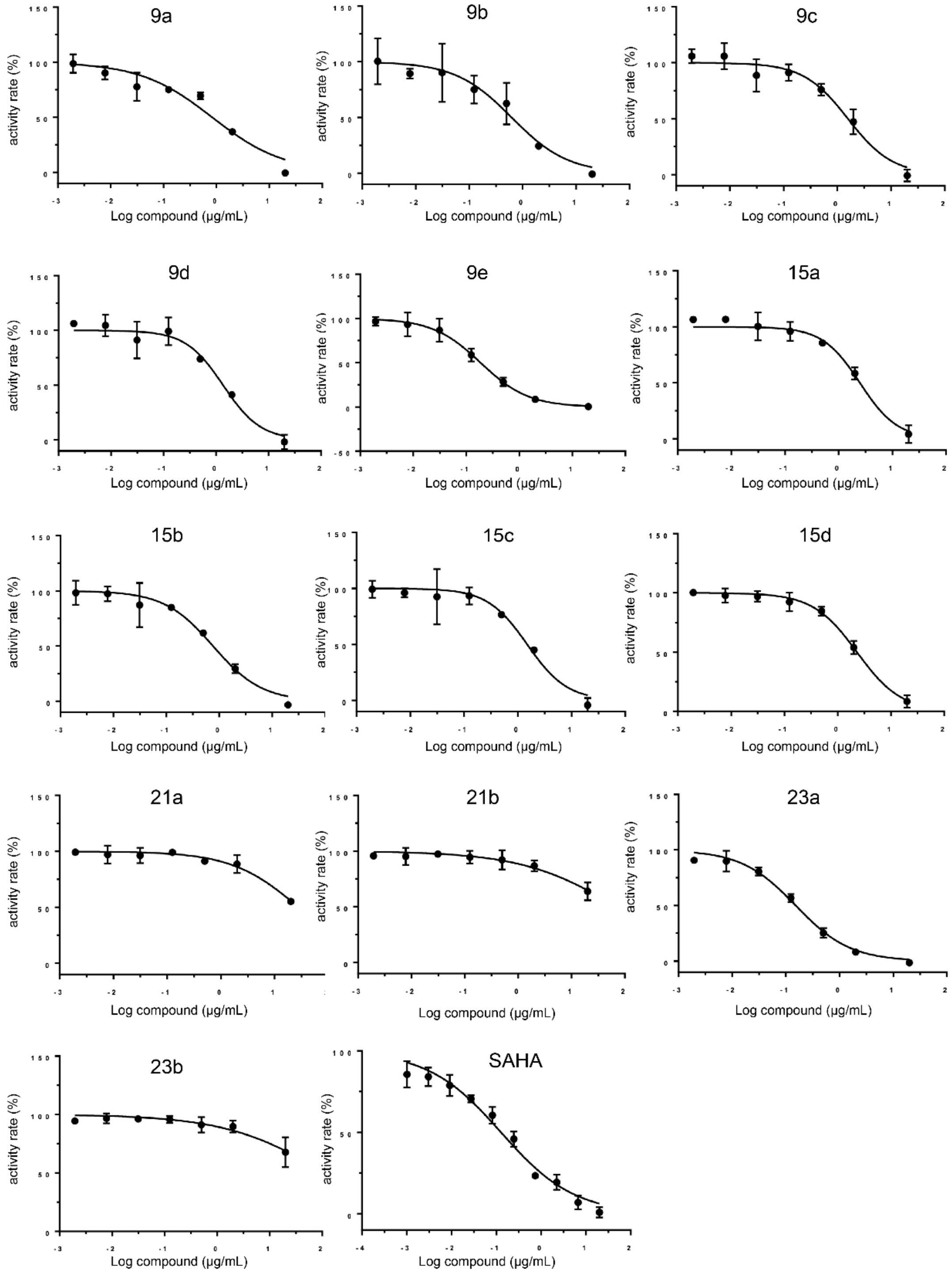

Figure S3. IC_{50} curve of synthesized PROTACs and SAHA against HDAC6.

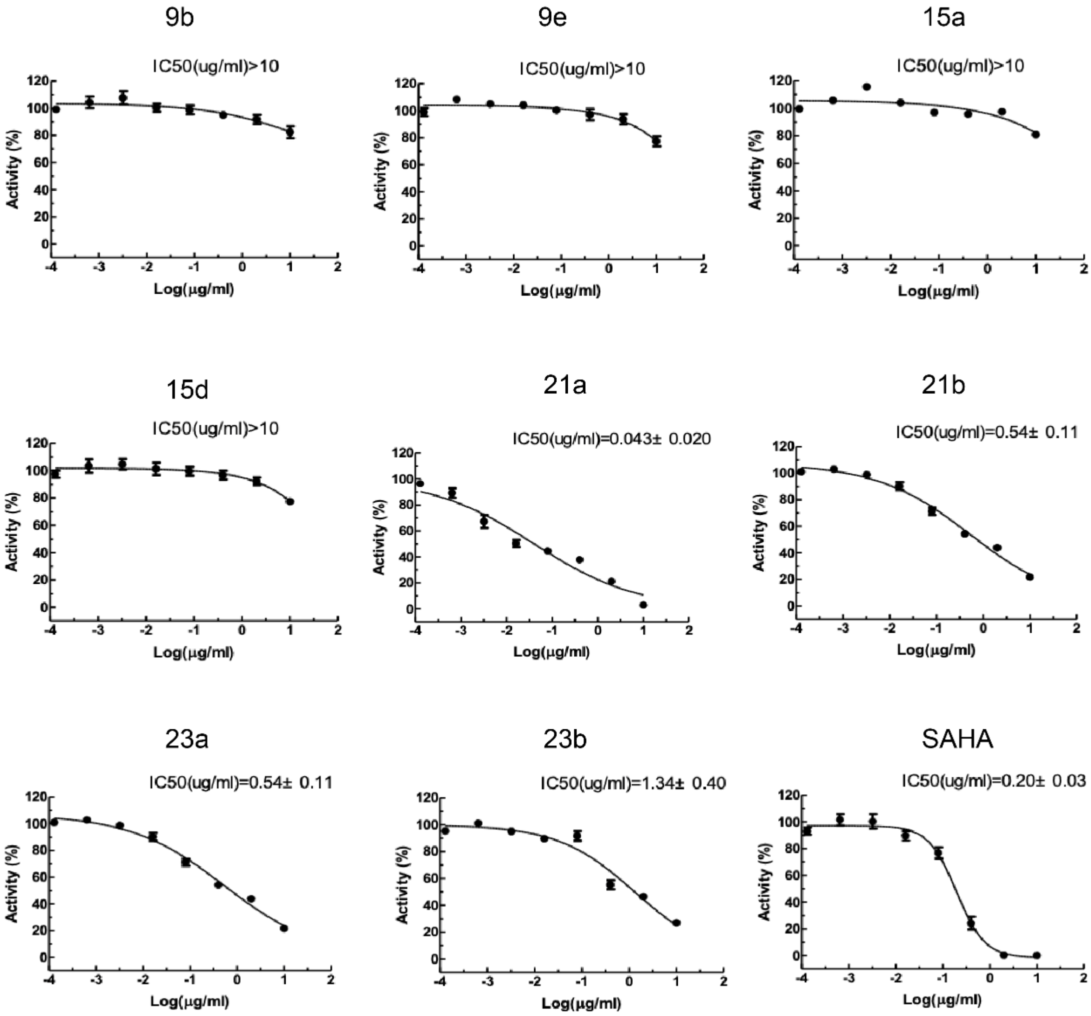

Figure S4. IC50 curve of synthesized PROTACs and SAHA against MM.1S cell line.

^{1}H-NMR for **9a**

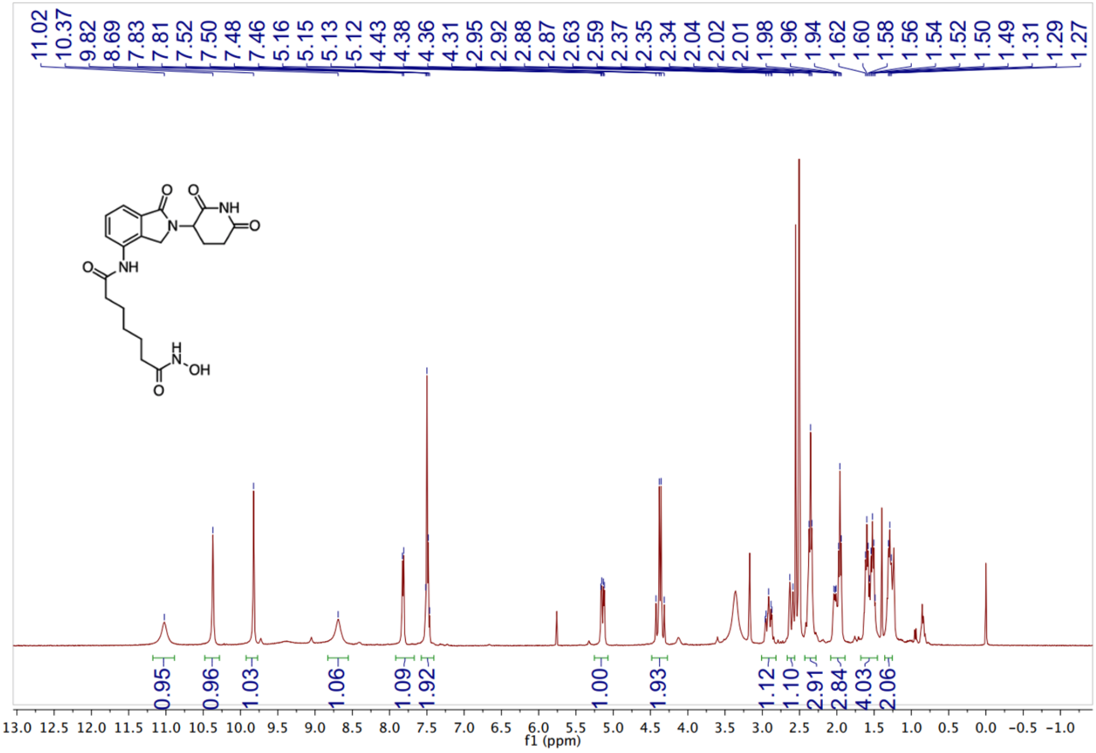

^{13}C-NMR for **9a**

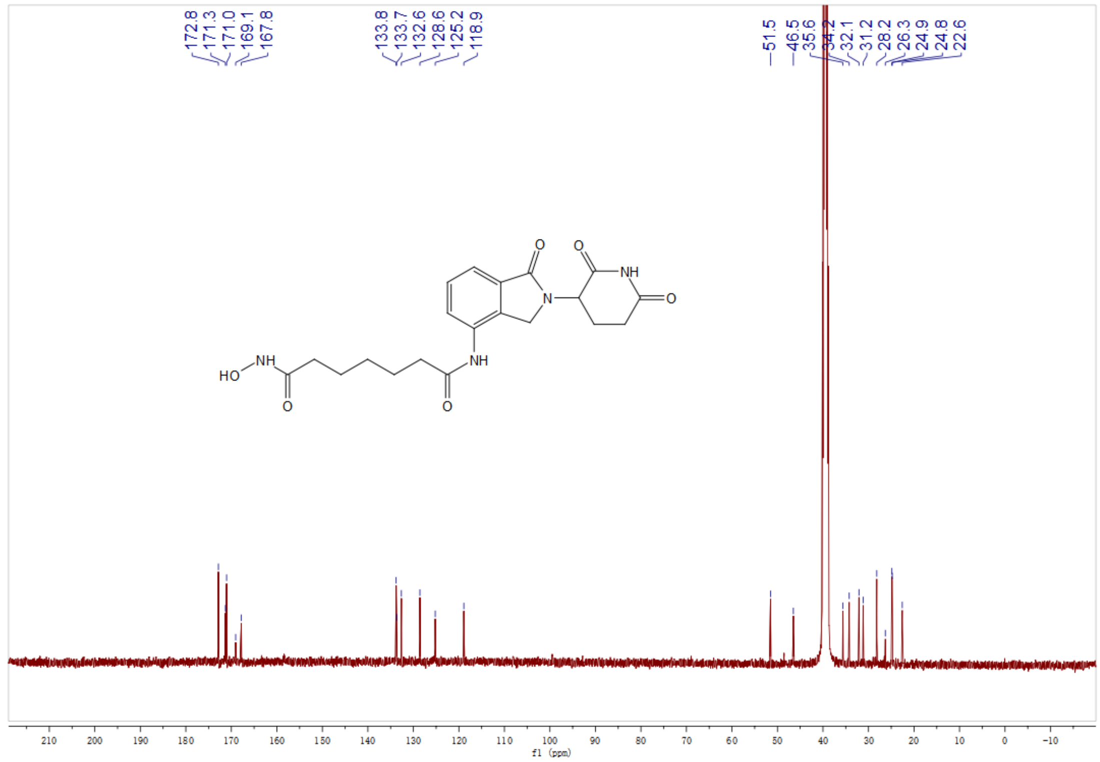

^{1}H-NMR for **9b**

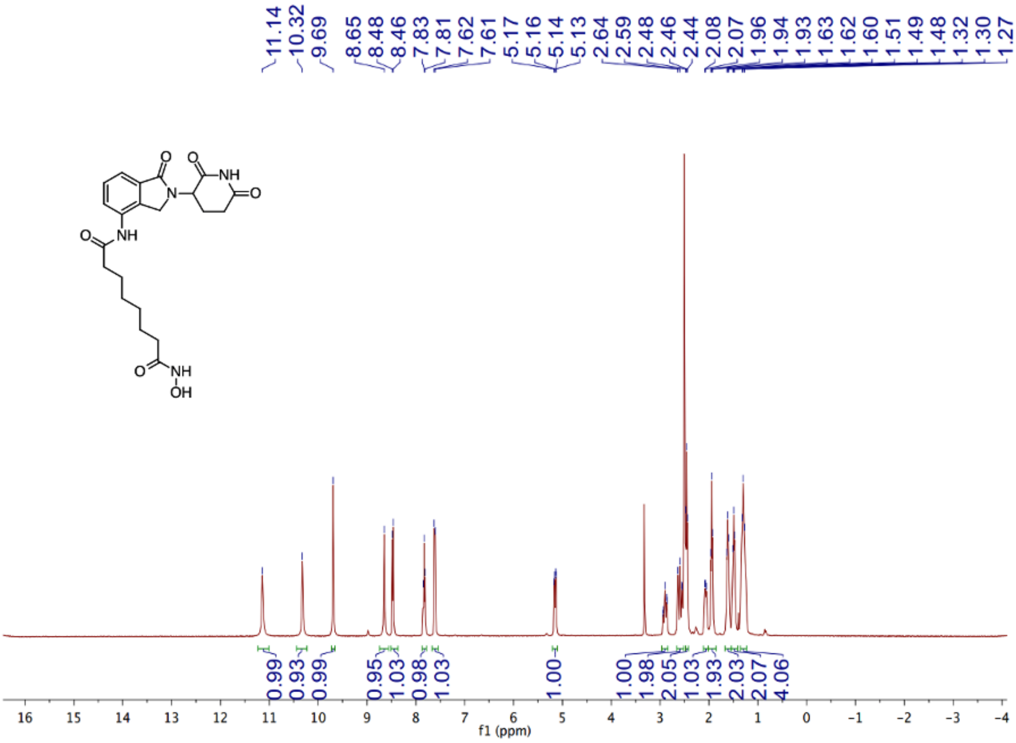

^{13}C-NMR for **9b**

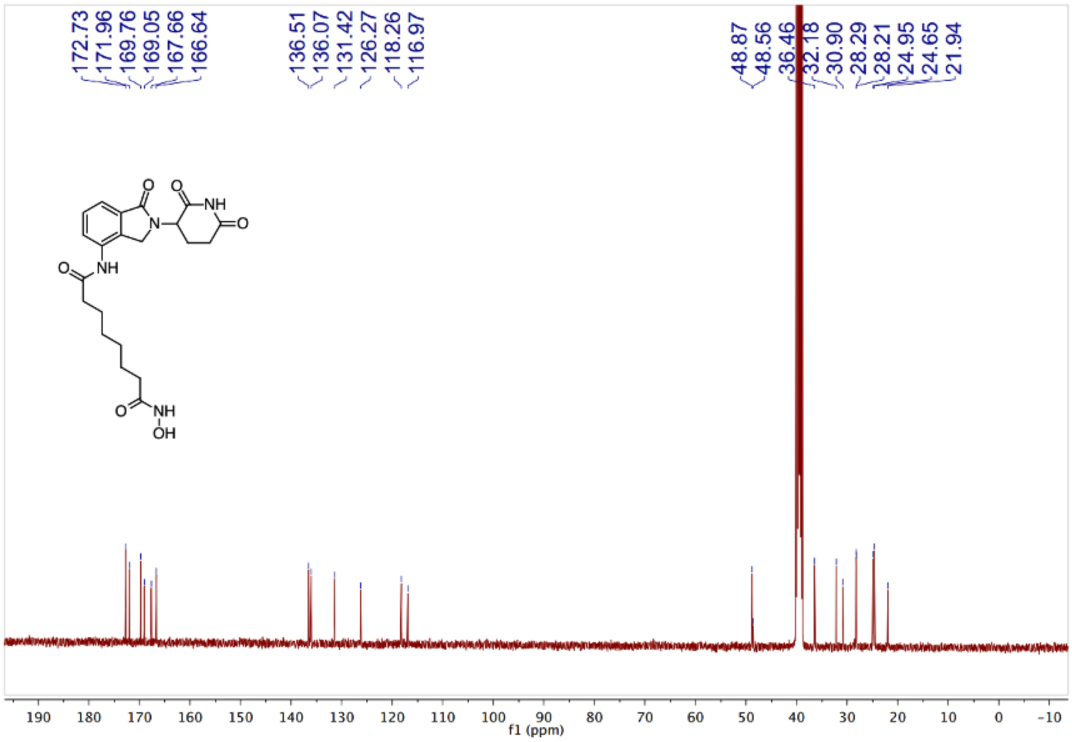

^{1}H-NMR for **9c**

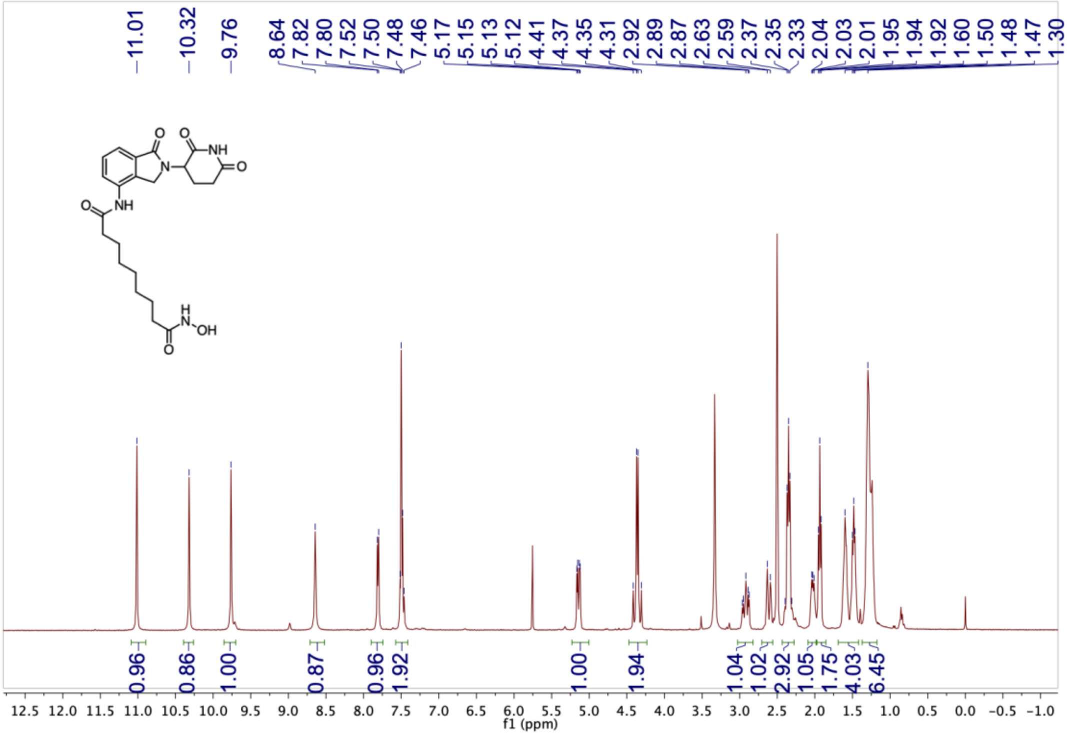

^{13}C-NMR for **9c**

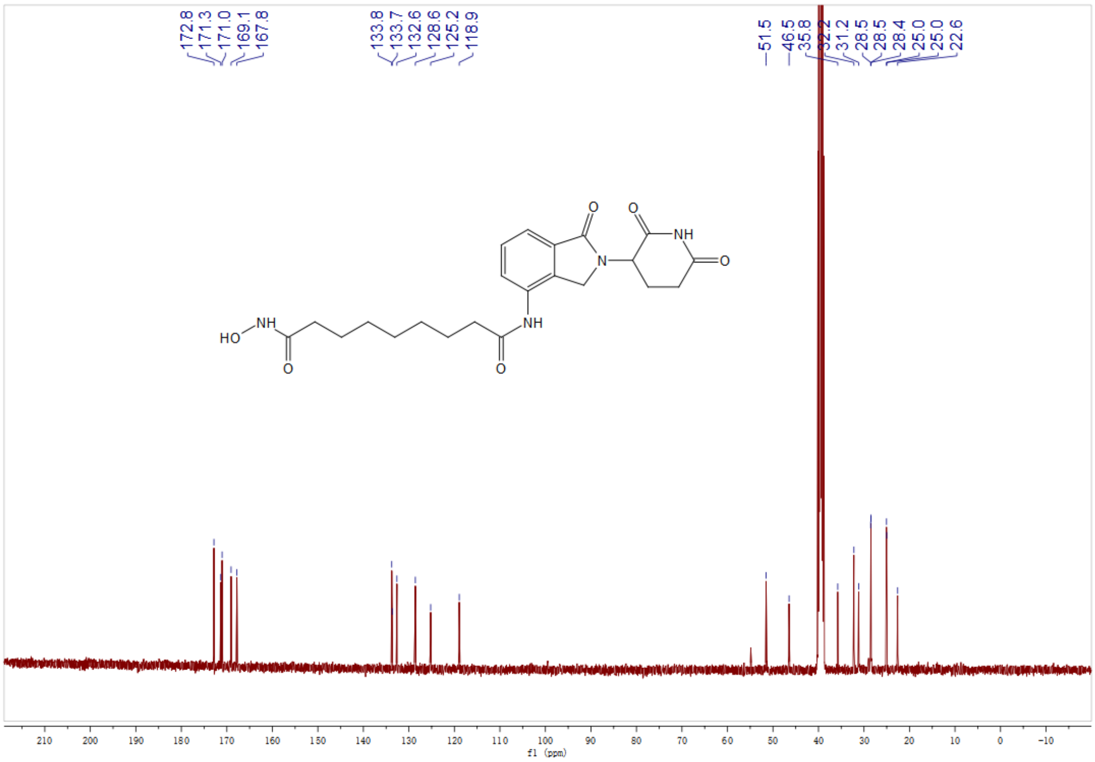

^{1}H-NMR for **9d**

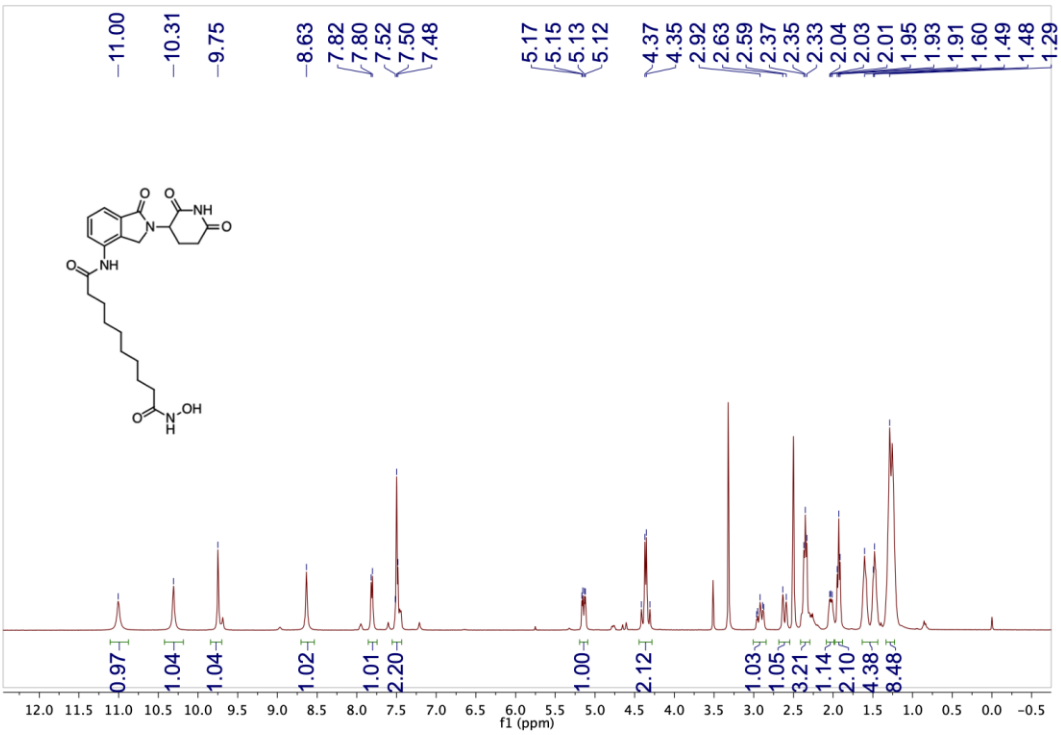

^{13}C-NMR for **9d**

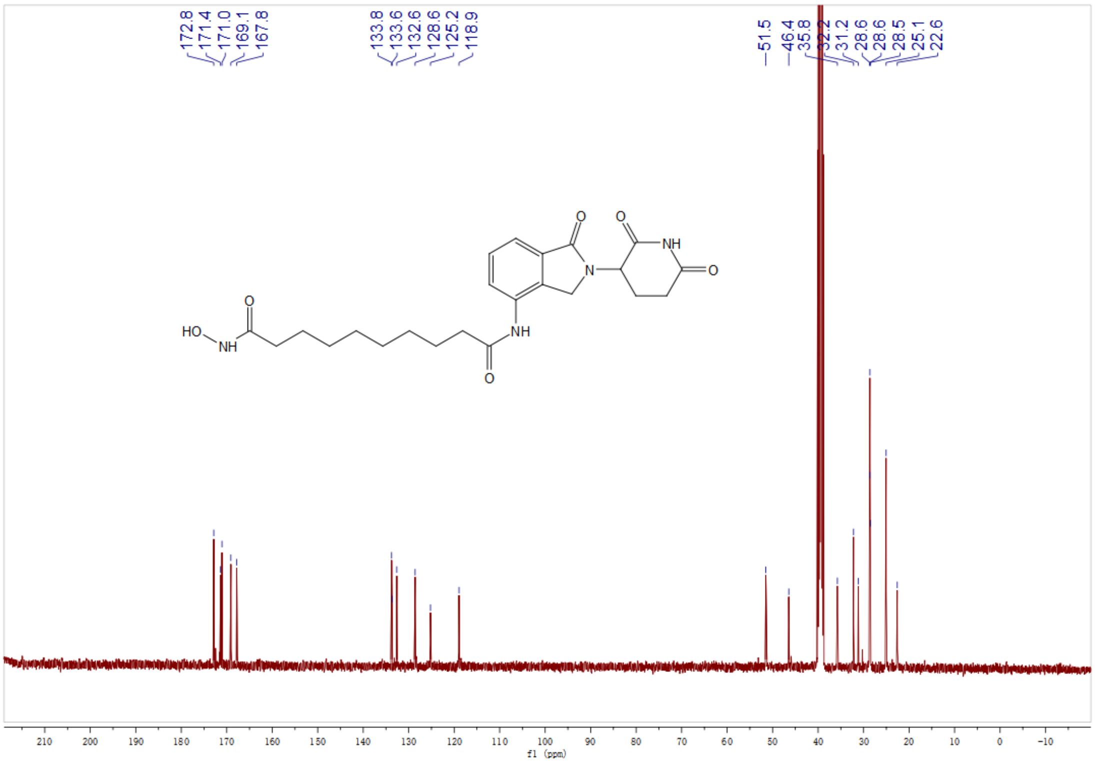

^{1}H-NMR for **9e**

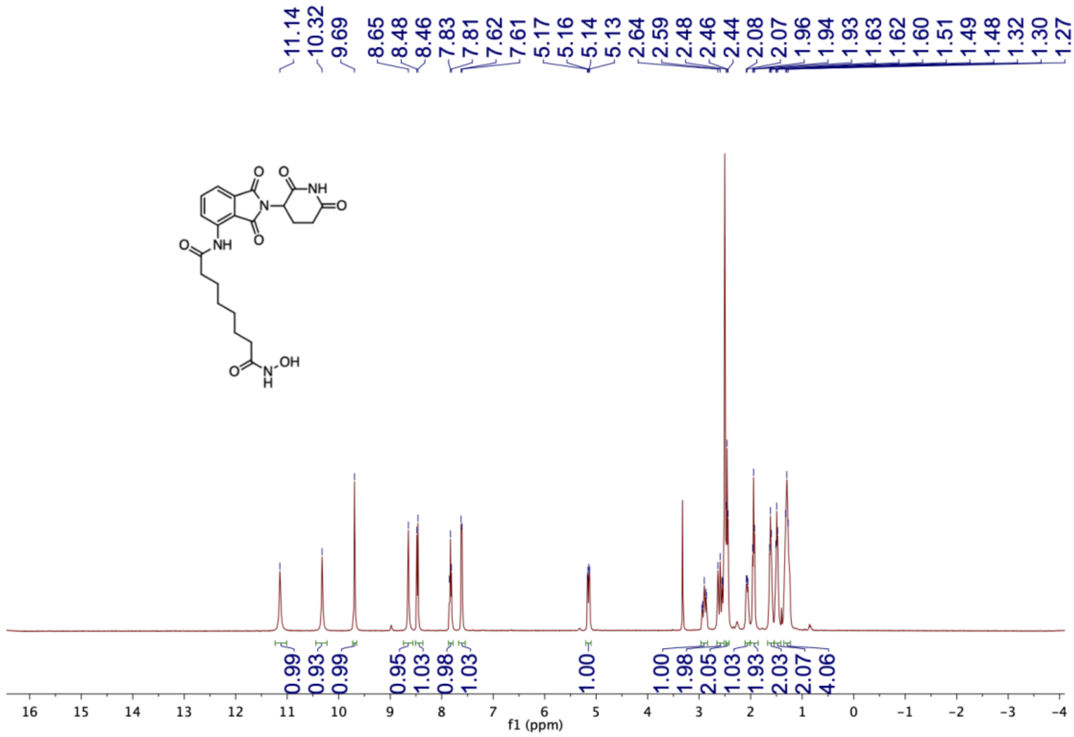

^{13}C-NMR for **9e**

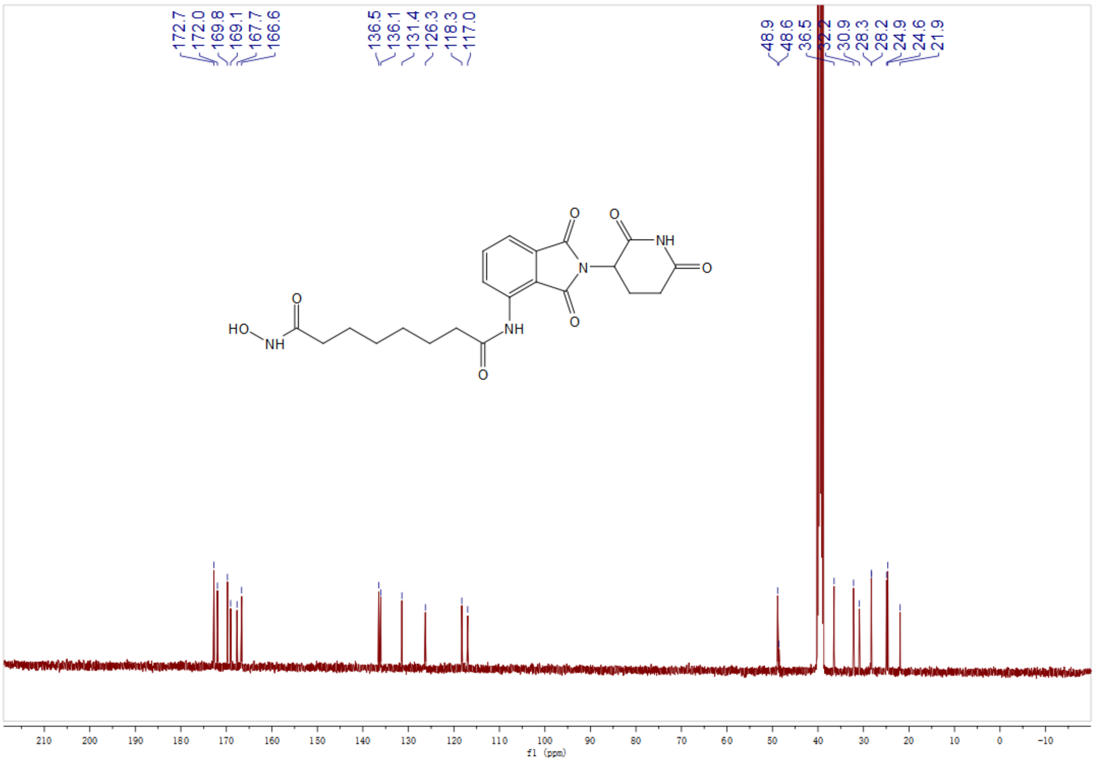

^{1}H-NMR for **15a**

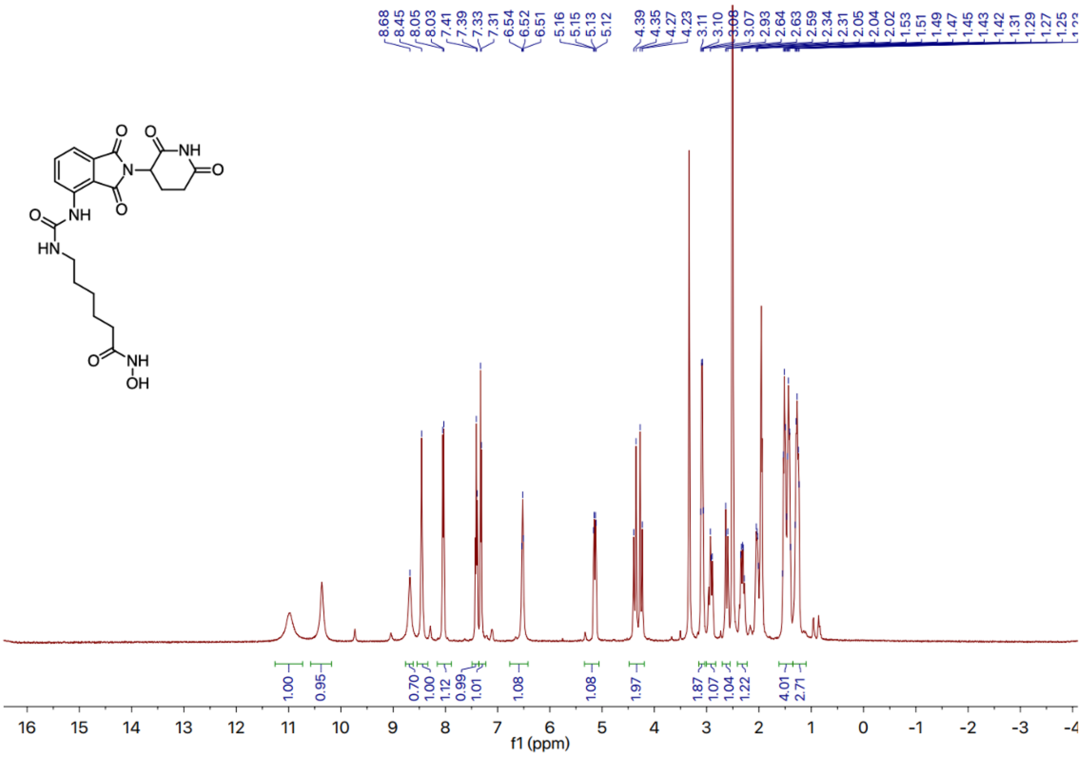

^{13}C-NMR for **15a**

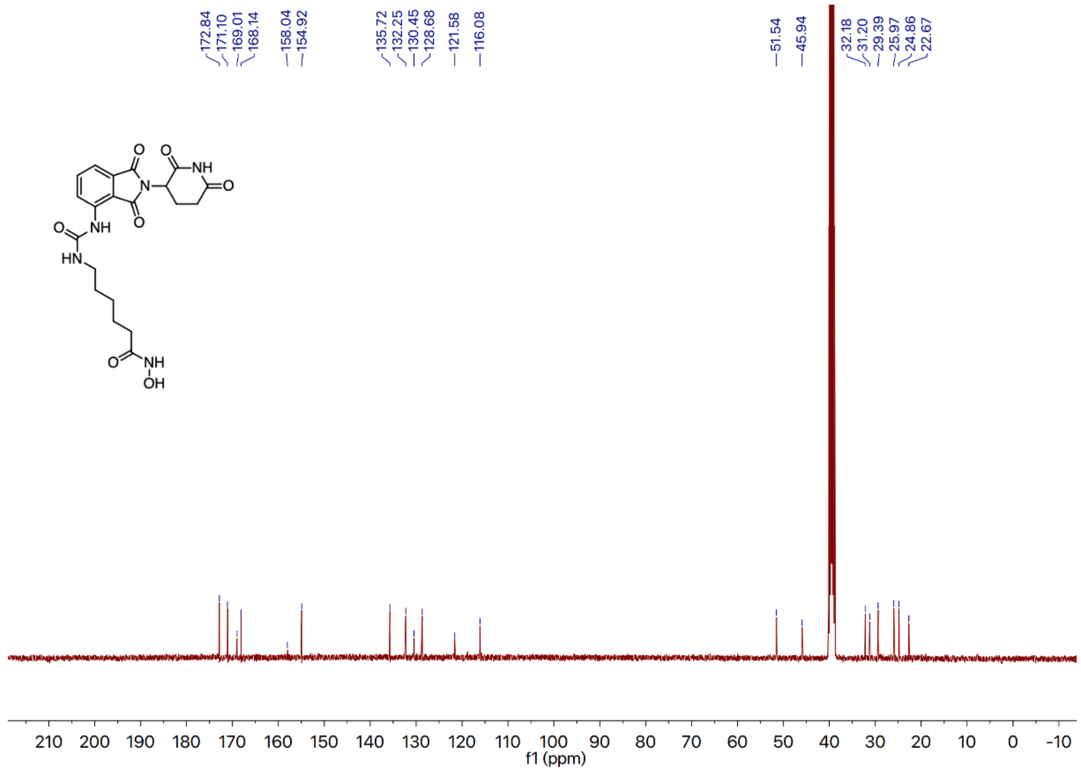

^{1}H-NMR for **15b**

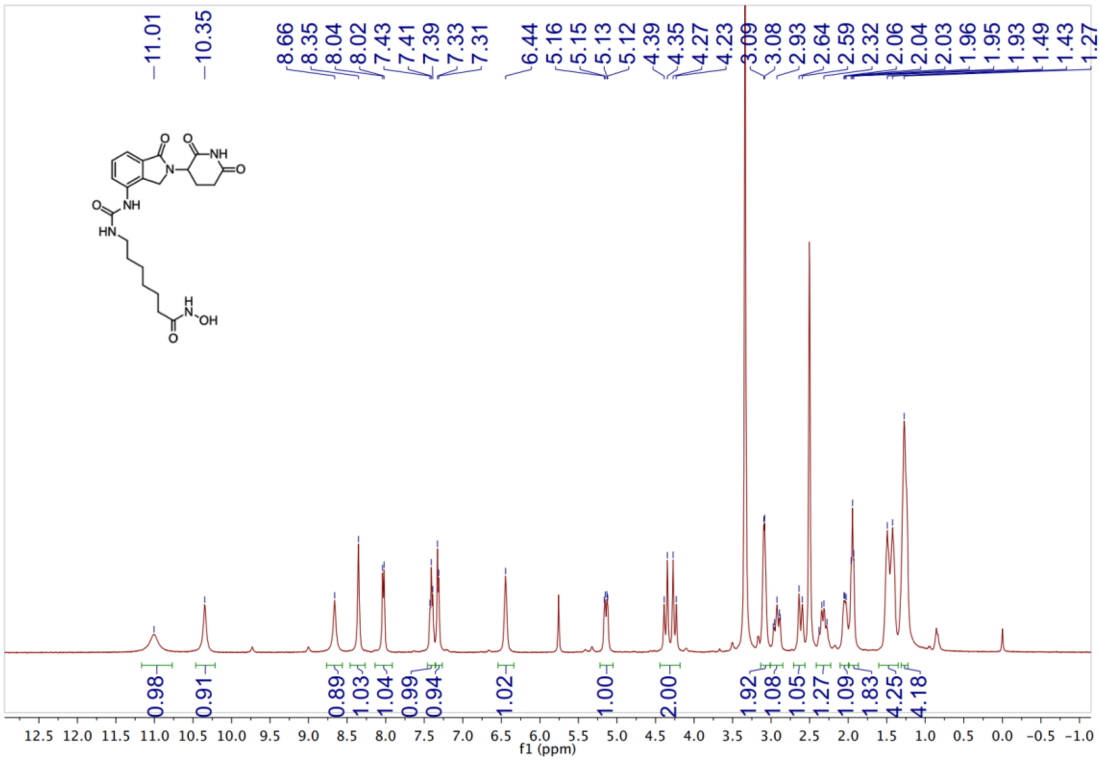

^{13}C-NMR for **15b**

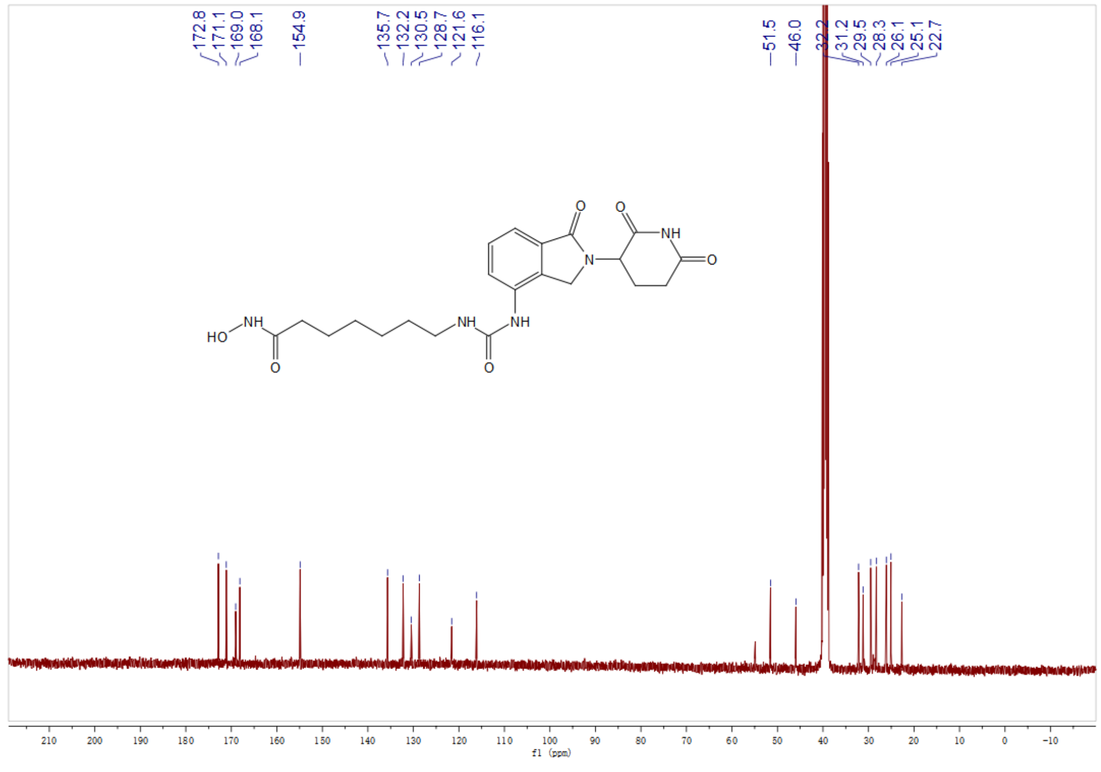

^{1}H-NMR for **15c**

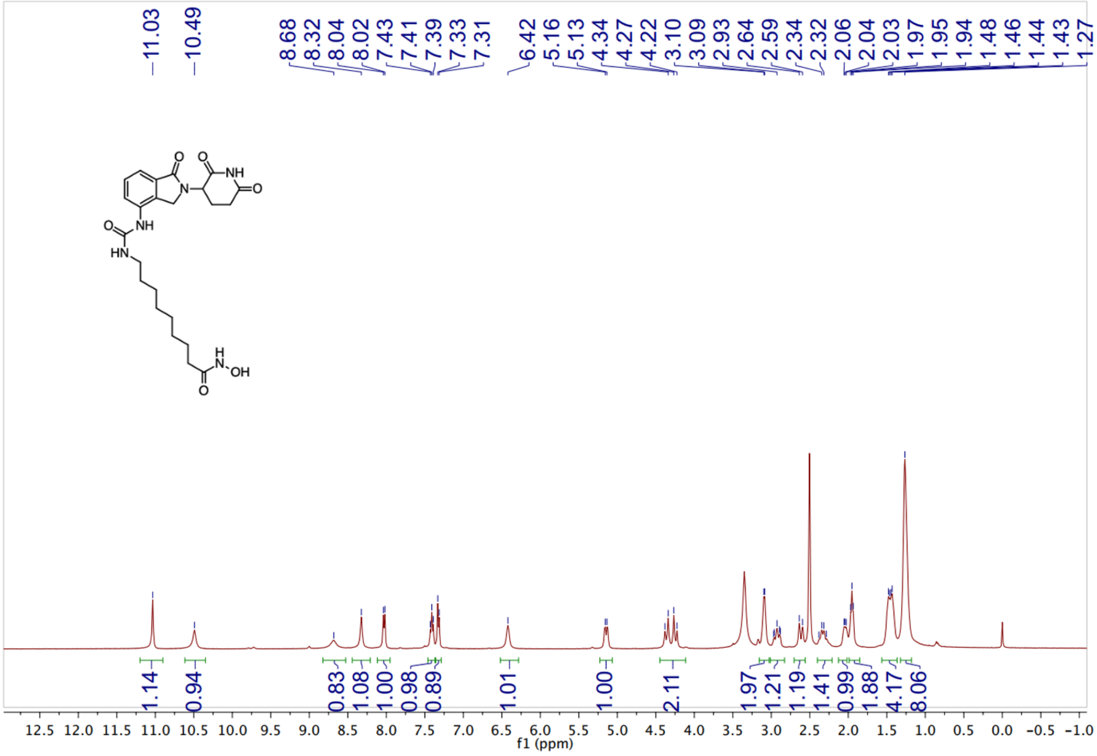

^{13}C-NMR for **15c**

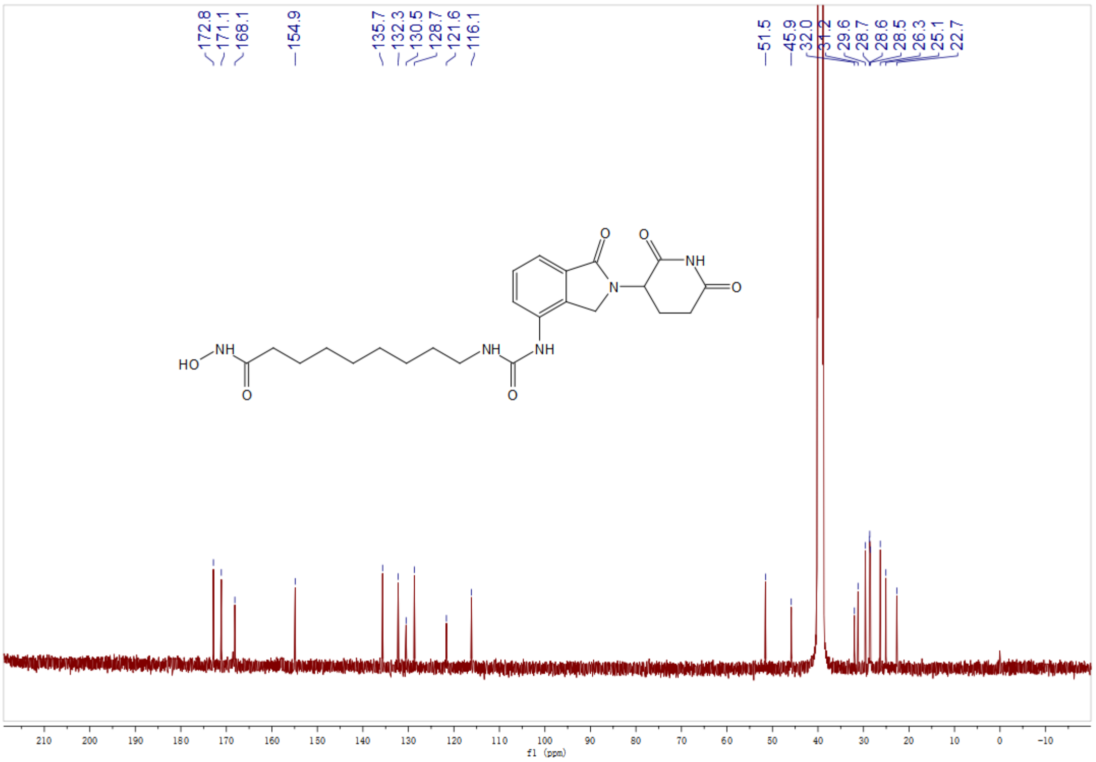

^{1}H-NMR for **15d**

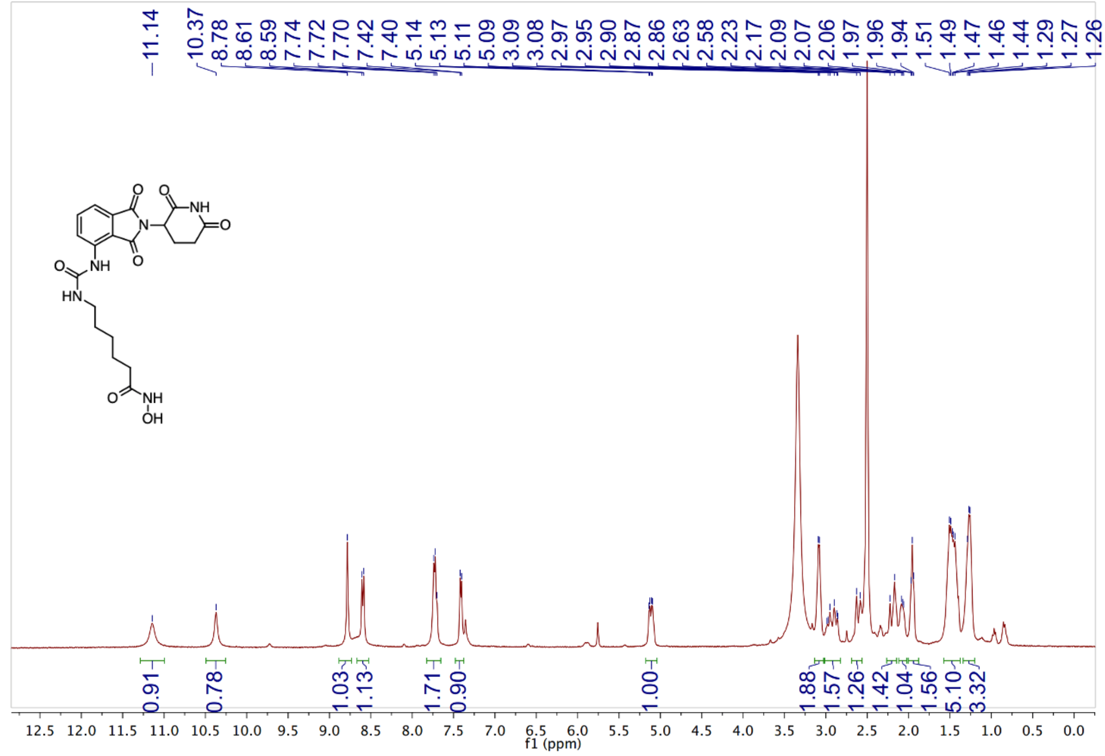

^{13}C-NMR for **15d**

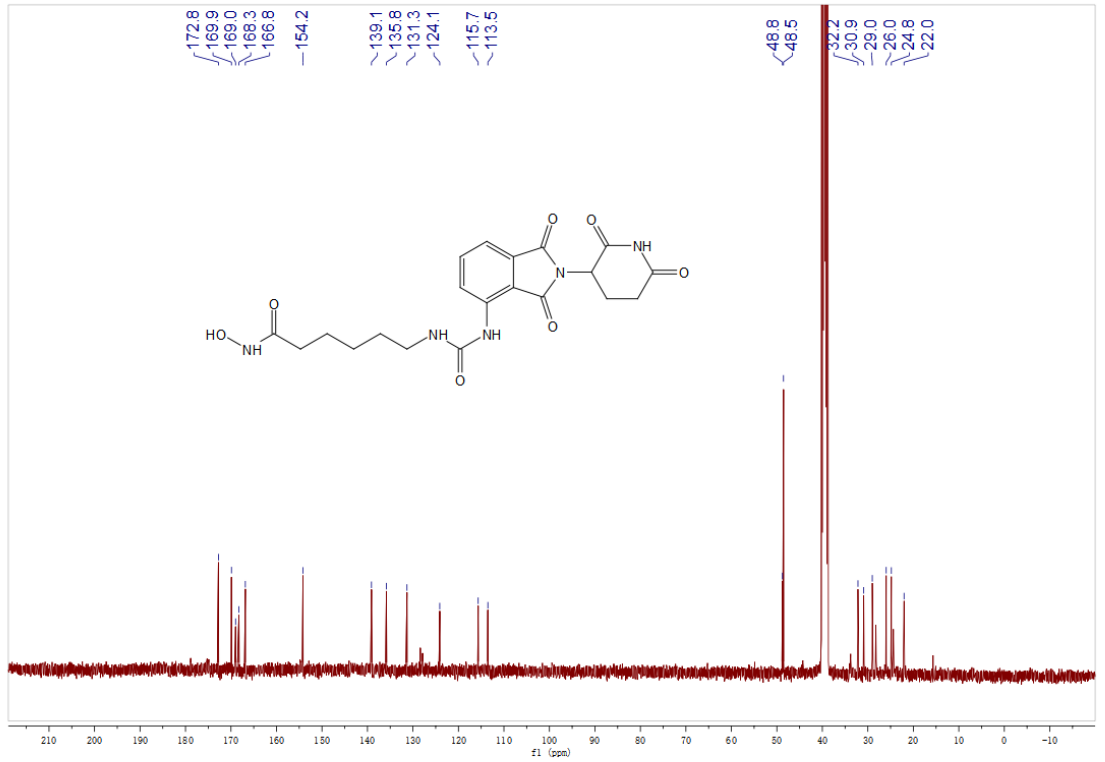

^{1}H-NMR for **21a**

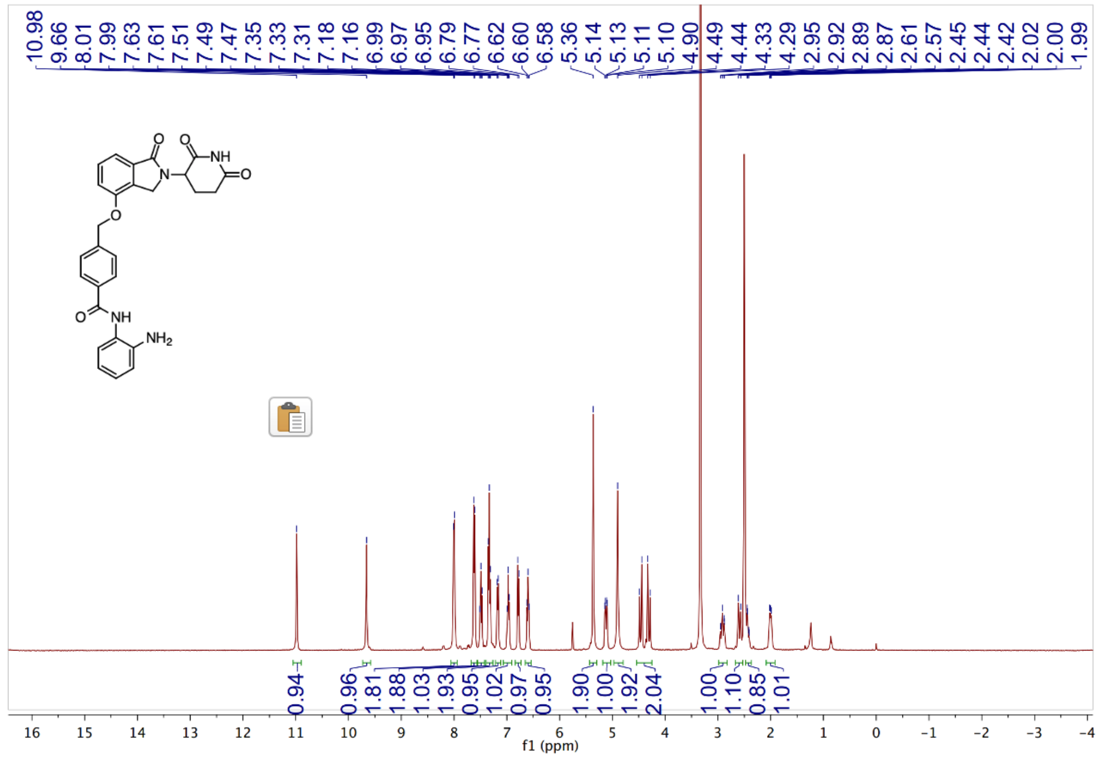

^{13}C-NMR for **21a**

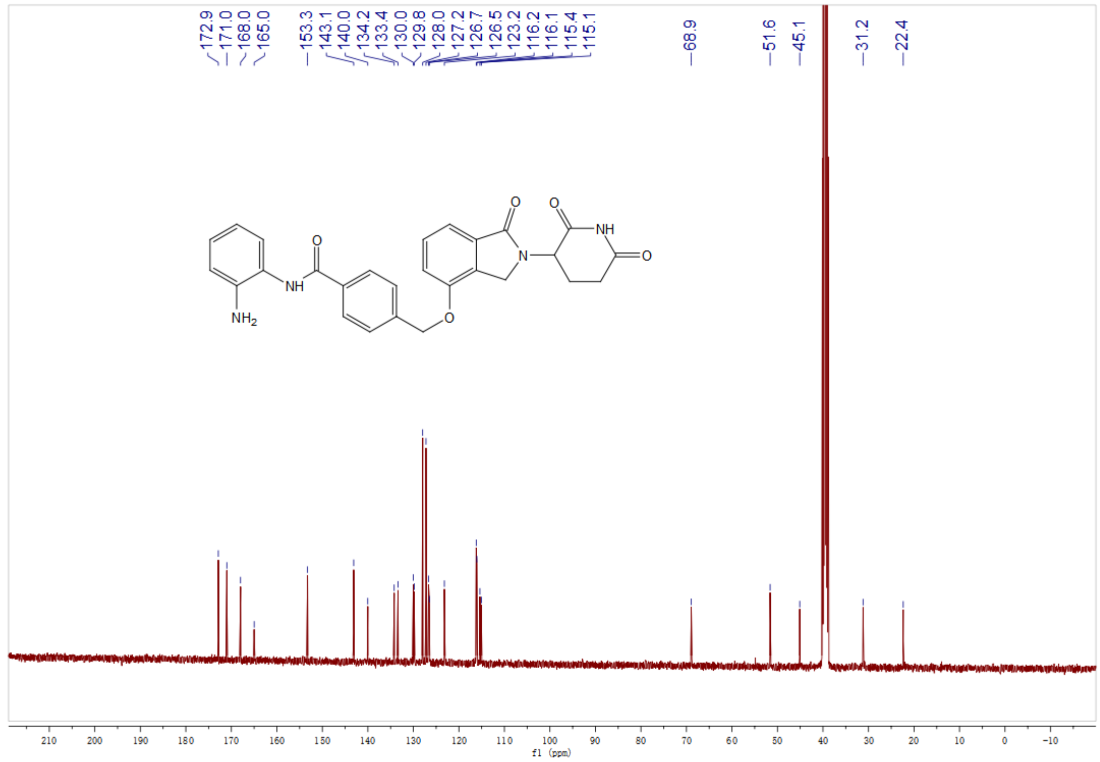

^{1}H-NMR for **21b**

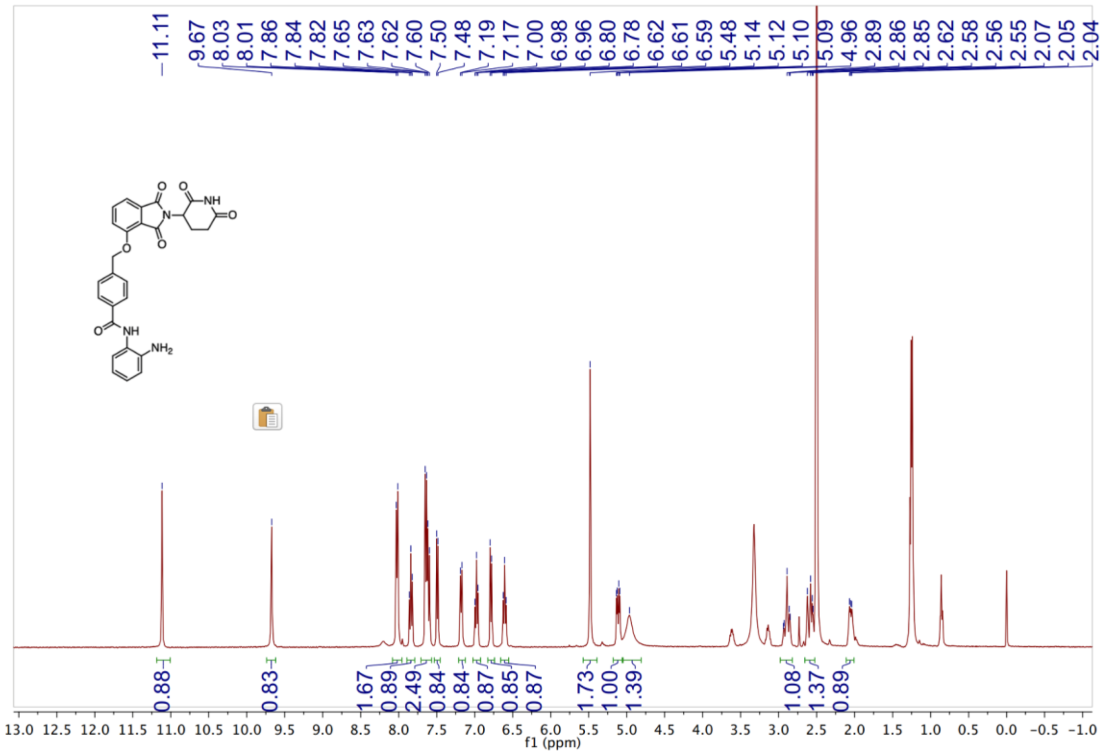

^{13}C-NMR for **21b**

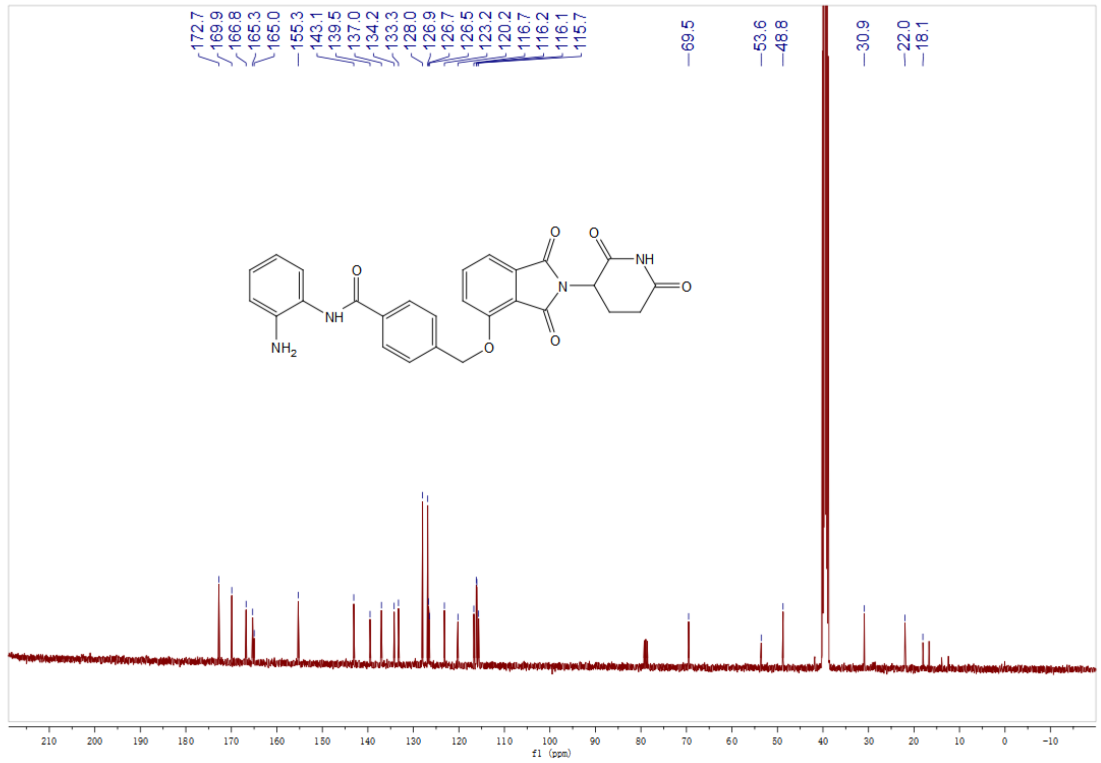

^{1}H-NMR for **23a**

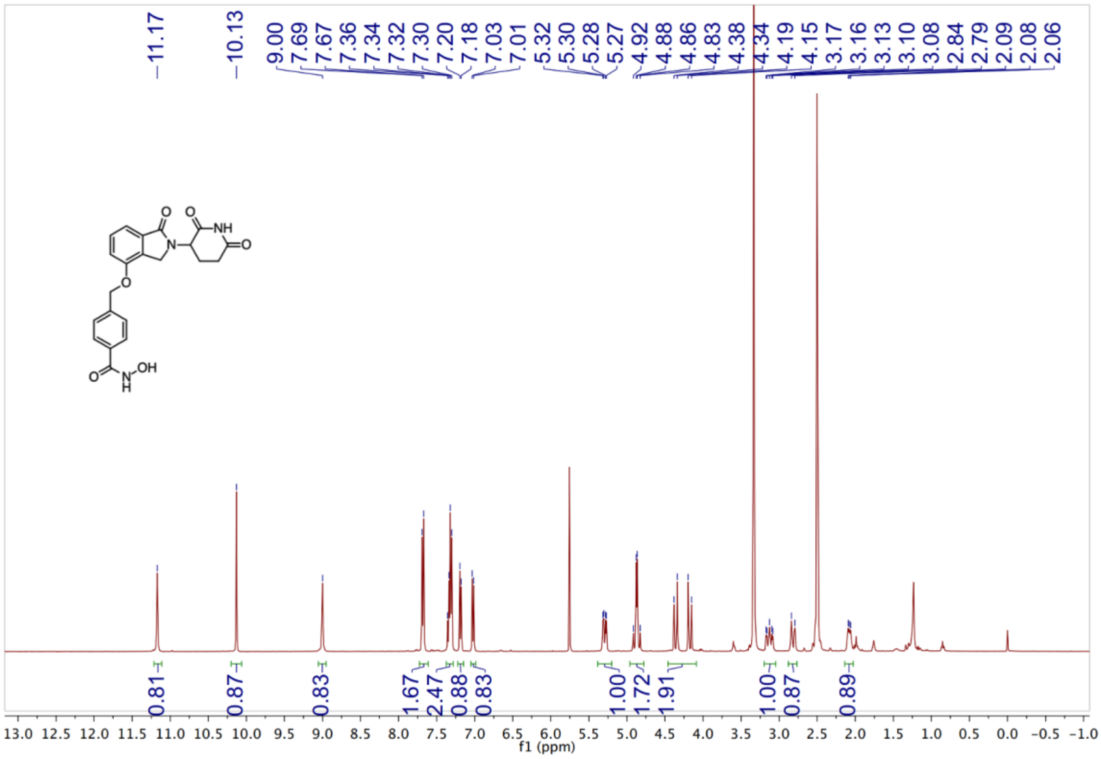

^{13}C-NMR for **23a**

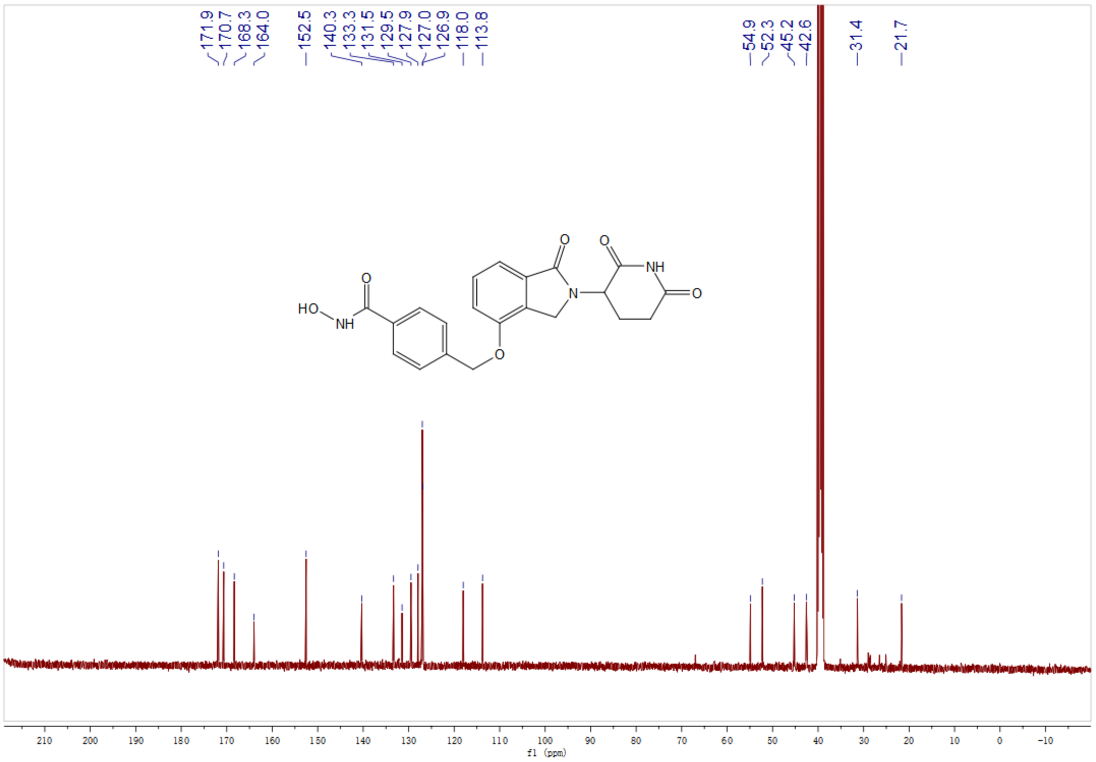

^{1}H-NMR for **23b**

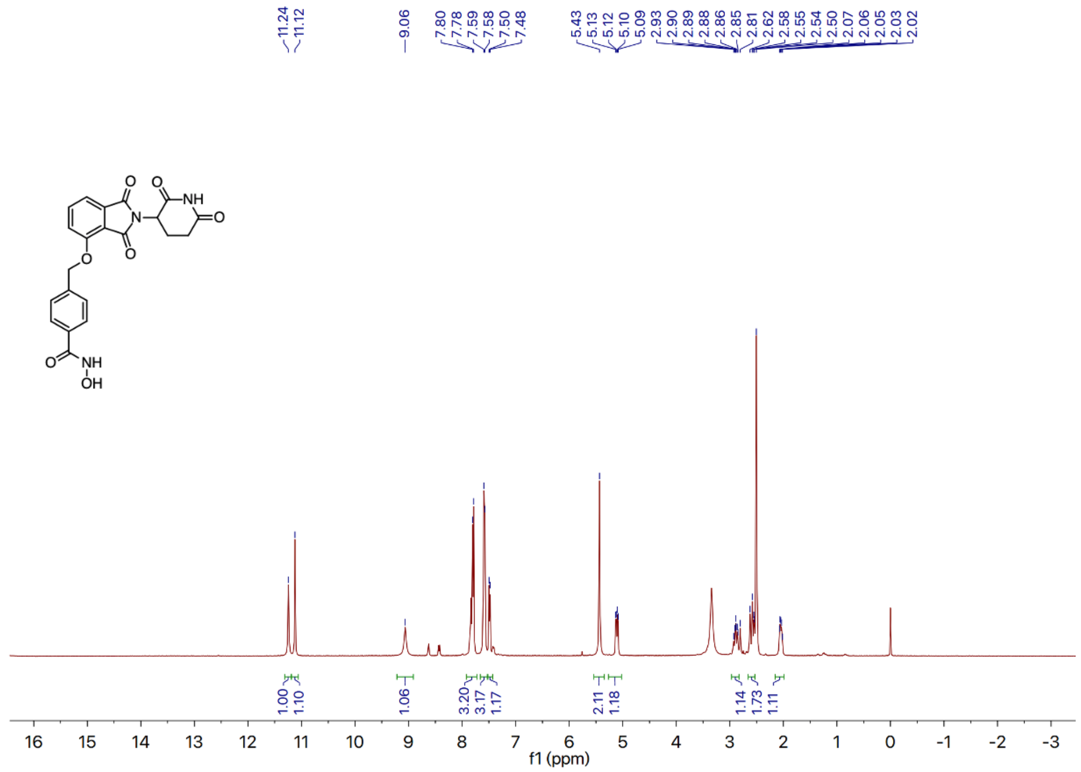

^{13}C-NMR for **23b**

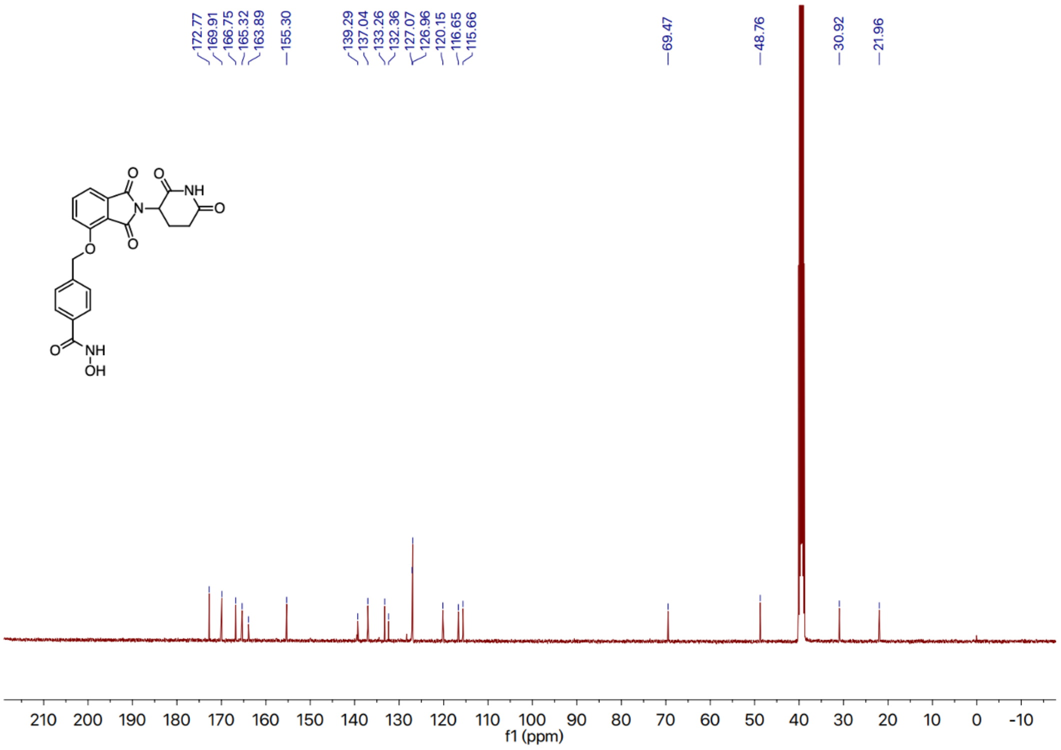

Supplement: Supplementary file 1 [file molecules-26-07241-s001.zip › molecules-1488850-supplementary.pdf]
